# Renal Lymphangiectasia: An Unusual Mimicker of Cystic Renal Disease – A Case Series and Literature Review

**DOI:** 10.7759/cureus.10849

**Published:** 2020-10-08

**Authors:** Shobana Umapathy, Ezhilmathi Alavandar, Rupa Renganathan, Thambidurai S, Venkatesh Kasi Arunachalam

**Affiliations:** 1 Radiology, Kovai Medical Center and Hospital, Coimbatore, IND

**Keywords:** lymphangiectasia, renal cyst, polycystic kidney disease, urinoma

## Abstract

Cystic renal lesions are commonly seen on a daily basis in abdominal imaging. Even though most cystic renal lesions are benign simple cysts, complex and multifocal cystic renal diseases are also common with a vast number of differentials. One of the rare mimickers of this condition is renal lymphangiectasia, and the disease can be diagnosed if radiologists are aware of the imaging findings, and this can help the physician to offer the appropriate treatment. We report a case series of five cases in our hospital and also review the literature on renal lymphangiectasia, including its pathophysiology, clinical presentation, imaging appearances, complications, treatment, and differentials.

## Introduction

Cystic renal lesions are routinely found during abdominal imaging. While most cystic renal lesions are benign simple cysts, complex and multifocal cystic renal diseases are also common with a vast number of differentials. One of the condition's rare mimickers is renal lymphangiectasia, and the disease can be diagnosed if radiologists are aware of the imaging findings, thereby aiding the physician to offer the appropriate treatment.

## Case presentation

Case series

Case 1

**Figure 1:**
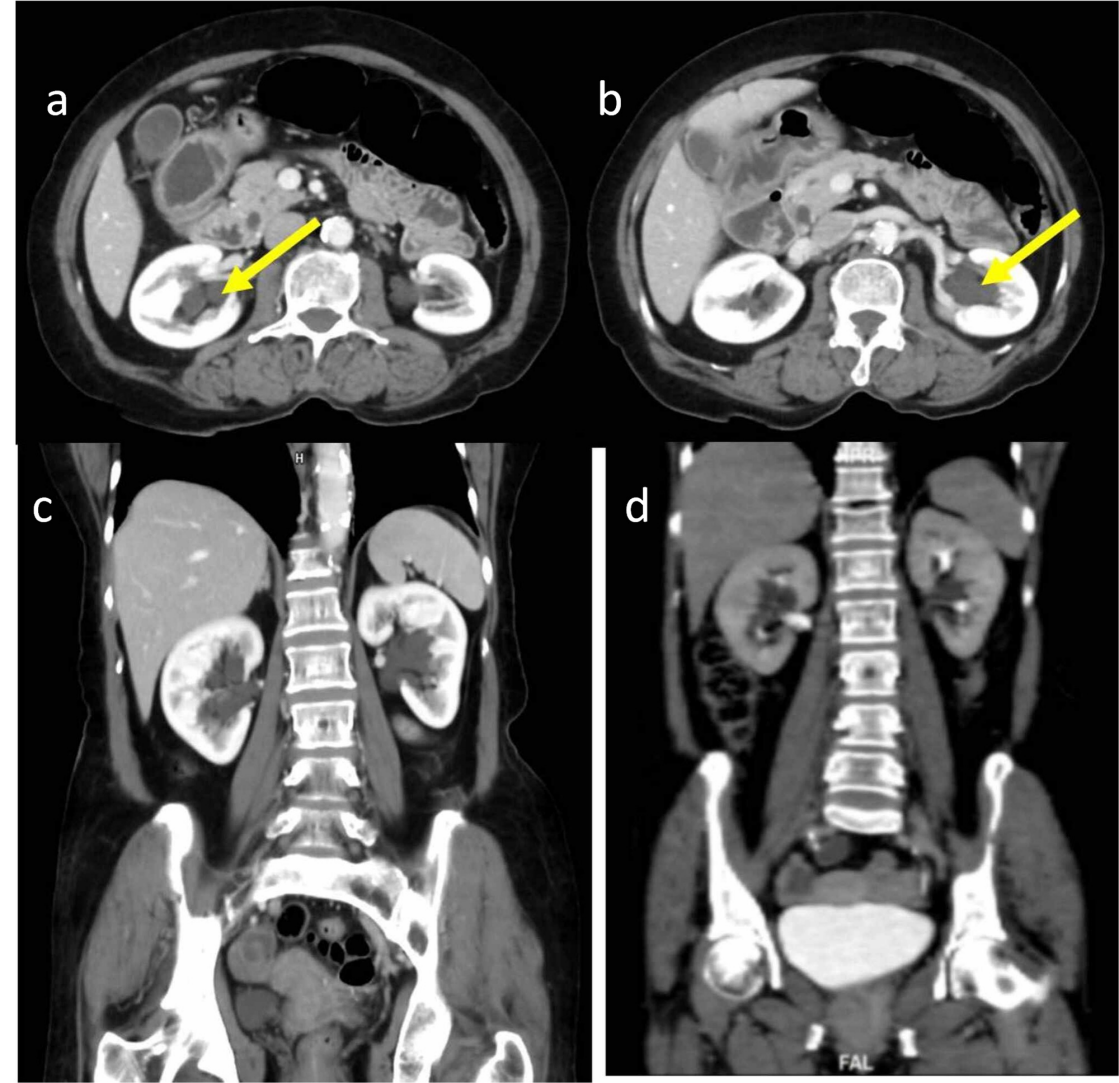
69-year-old female with bilateral peripelvic renal lymphangiectasia Contrast-enhanced CT of the abdomen in axial (a, b) and coronal (c) planes in the venous phase demonstrates lobulated low-density peripelvic lesions (yellow arrows) in both kidneys splaying the collecting system. Coronal contrast-enhanced CT of the abdomen in the delayed phase in the coronal plane (d) demonstrates no opacification of contrast in the low-density peripelvic lesions CT: computed tomography

A 69-year-old female presented with bilateral abdominal pain for 15 days. Ultrasound abdomen revealed anechoic fluid-filled avascular cystic spaces insinuating the bilateral renal sinuses. The patient underwent a contrast-enhanced CT scan of the abdomen. The contrast study revealed lobulated low-density peripelvic lesions in both kidneys splaying the collecting system (Figure 1). There was no contrast opacification of the lesions on delayed scans in the excretory phase. There was no other abnormality in the rest of the abdomen. The patient was managed conservatively.

Case 2

**Figure 2:**
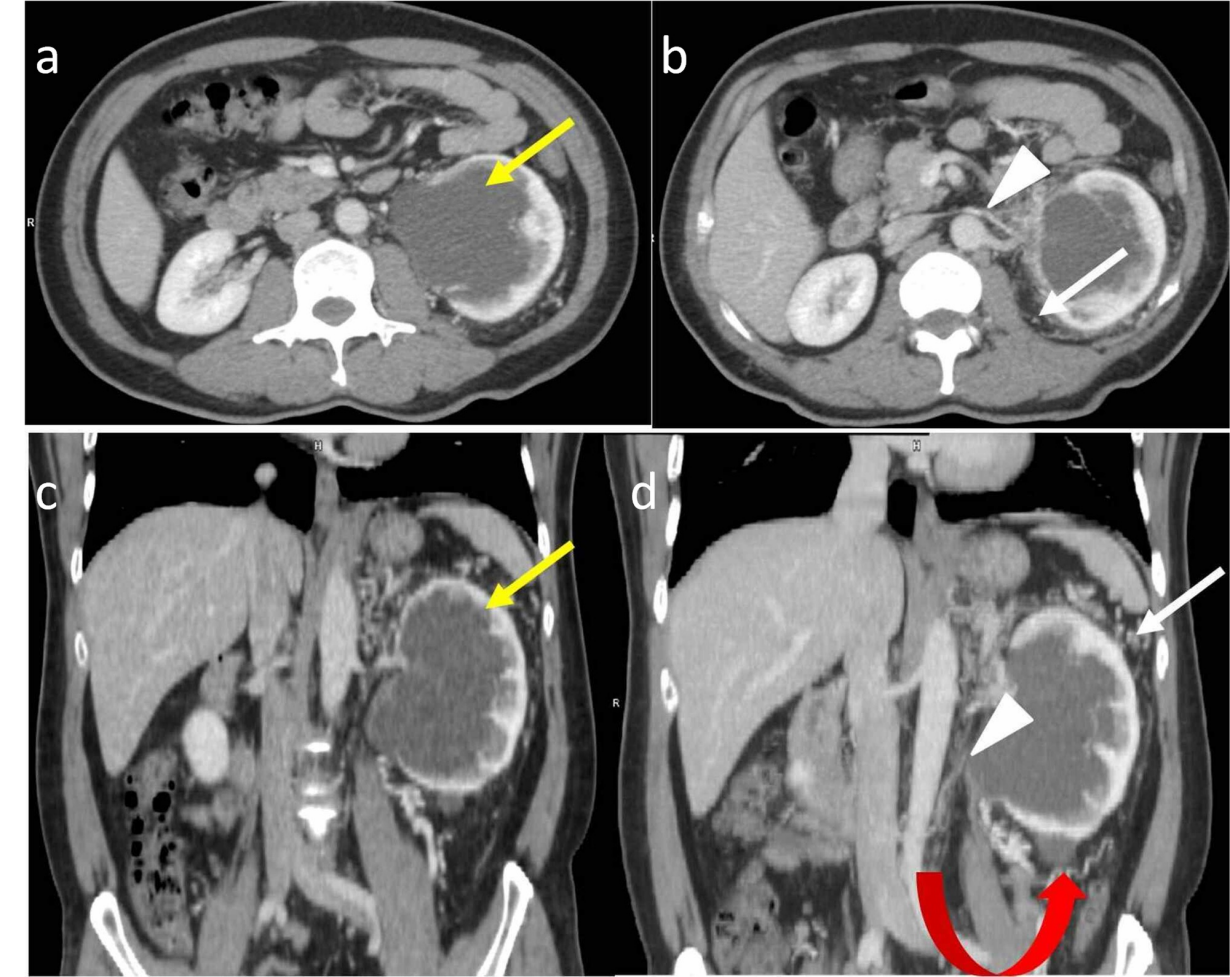
49-year-old male with left peripelvic renal lymphangiectasia Contrast-enhanced CT of the abdomen in the venous phase in axial (a, b) and coronal planes (c, d) demonstrates a peripelvic cystic lesion (yellow arrows) on the left side. It also demonstrates the attenuated caliber of the left renal vein (white arrowheads) with perirenal collaterals (white arrows) consistent with chronic left renal vein thrombosis. Associated Bosniak type I cyst (red curved arrow) is seen in the lower pole of the left kidney CT: computed tomography

**Figure 3:**
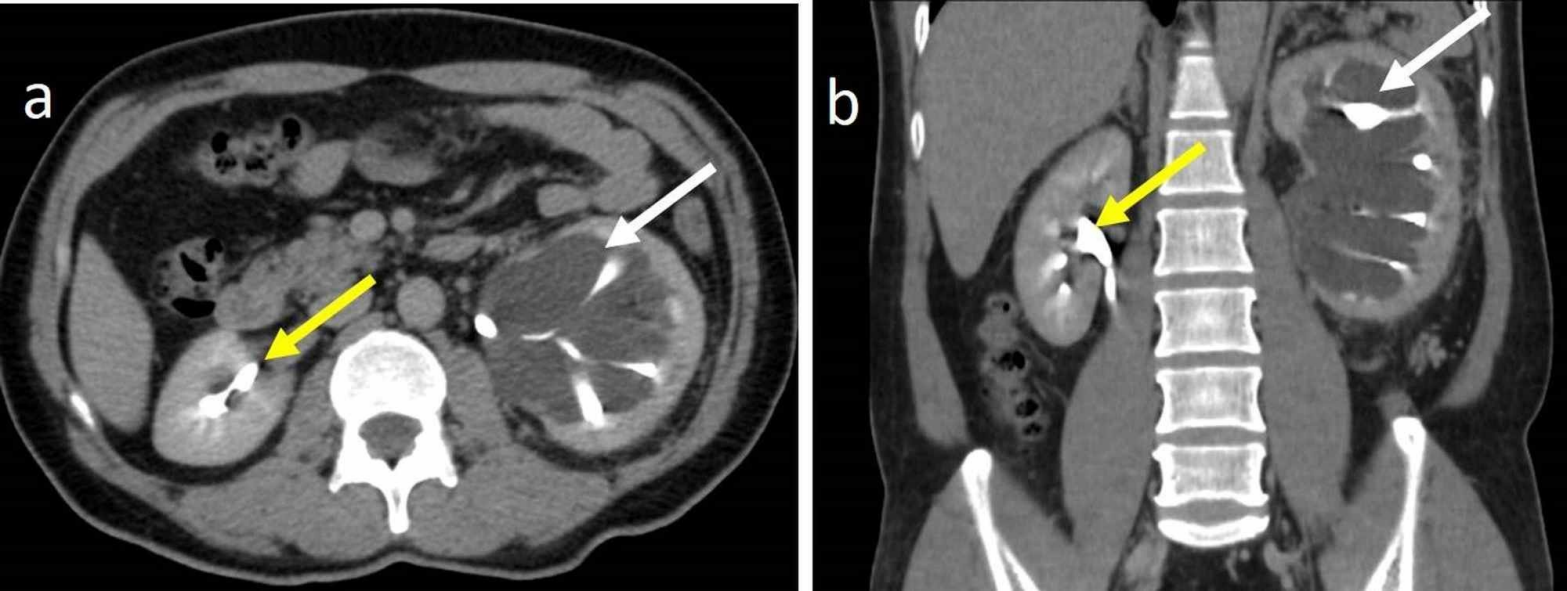
49-year-old male with left peripelvic renal lymphangiectasia (same patient as in Figure 2) Axial (a) and coronal (b) contrast-enhanced CT of the abdomen in the delayed phase demonstrates normal excretion of contrast with splaying of the pelvicalyceal system (white arrow) and non-opacification of peripelvic cystic lesion on the left side. The pelvicalyceal system on the right side (yellow arrow) is normal CT: computed tomography

A 49-year-old male presented with left loin pain for one month. The patient underwent a contrast-enhanced CT scan of the abdomen. The CT images revealed a large cystic lesion with an imperceptible wall measuring 9 x 6.5 x 6.2 cm (CC x TR x AP) in the left renal sinus region indenting the adjacent parenchyma, distorting the pelvicalyceal system with associated extensive perinephric fat stranding. A simple cortical cyst measuring 1.8 x 1.7 cm was also noted in the lower pole of the left kidney. The left renal vein was smaller in caliber but showed opacification with contrast. Multiple perirenal collaterals were seen (Figures 2, 3). The features were secondary to chronic left renal vein thrombosis. The rest of the abdominal organs were normal. Imaging features were suggestive of peripelvic renal lymphangiectasia on the left side with features of chronic left renal vein thrombosis. The patient was properly diagnosed on a CT scan and the other differential diagnoses were excluded. The patient was managed conservatively.

Case 3

**Figure 4:**
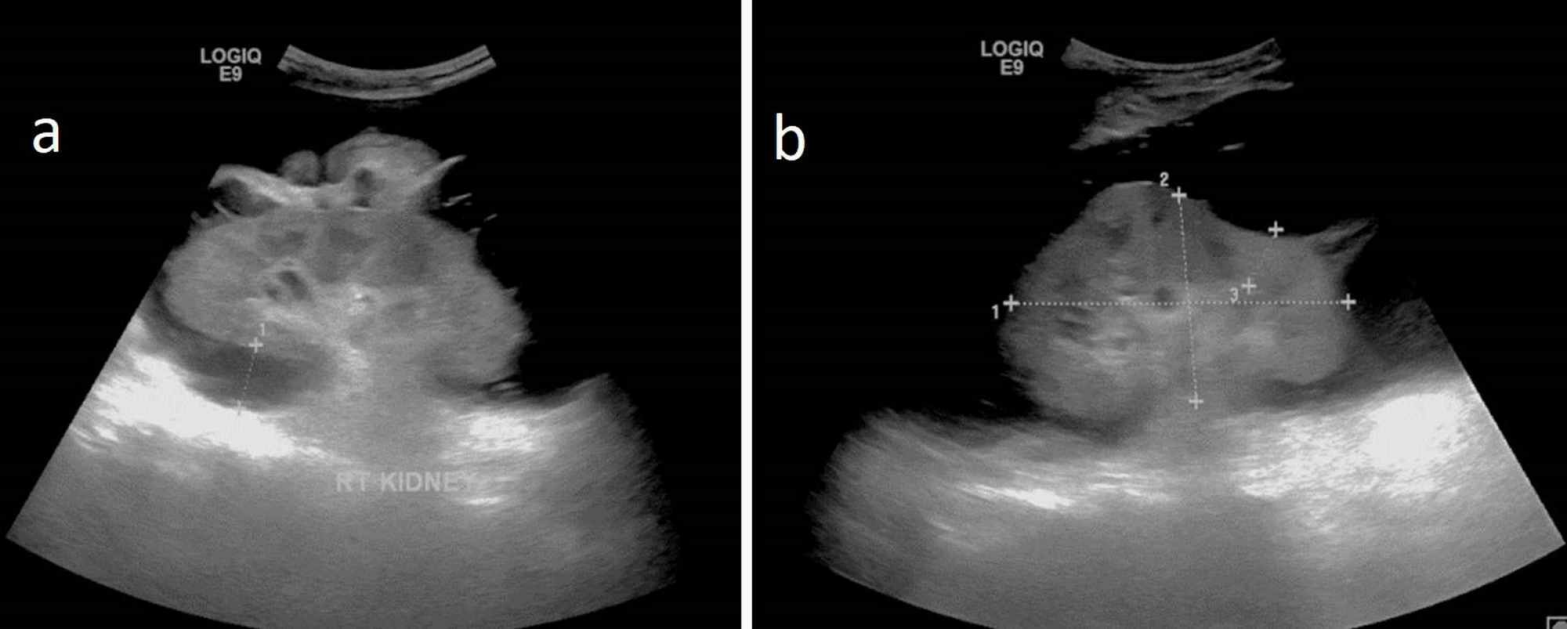
43-year-old male – a case of Budd-Chiari syndrome with bilateral perinephric renal lymphangiectasia Greyscale ultrasound in the oblique sagittal plane of both kidneys (a: right kidney, b: left kidney) showing anechoic collection with internal septations in the perinephric region on both sides

A 43-year-old male with a past medical history of Budd-Chiari syndrome and inferior vena cava (IVC) stenting presented with back pain, abdominal discomfort, and distention for three days. Ultrasound abdomen images revealed raised renal cortical echoes with altered corticomedullary differentiation. Bilateral anechoic perinephric collection with internal septations was noted surrounding both kidneys (Figure 4). Altered liver parenchymal echoes and patent IVC stent were also noted.

**Figure 5:**
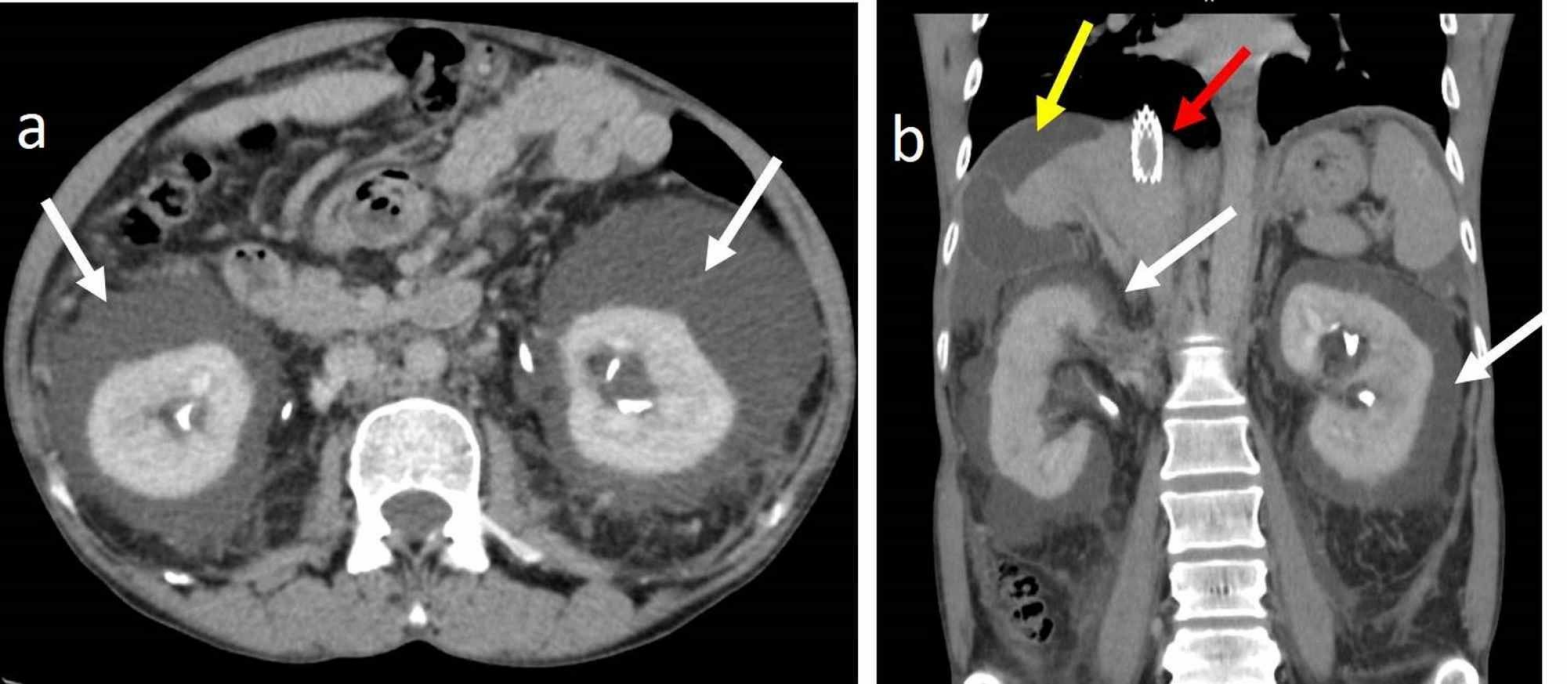
43-year-old male with bilateral perinephric renal lymphangiectasia (same patient as in Figure 4) Axial (a) and coronal (b) contrast-enhanced CT of the abdomen in the delayed phase demonstrates bilateral perinephric fluid collections (white arrows). The coronal image also demonstrates ascites (yellow arrow) and IVC stent (red arrow) CT: computed tomography; IVC: inferior vena cava

Contrast-enhanced CT scan images of the abdomen showed circumferential perinephric fluid collections on both sides with anterior displacement of anterior pararenal fascia (Figure 5). Extensive perinephric fat stranding with few small fluid collections in the pararenal space was also made out. Minimal ascites was also noted. The perinephric fluid collection, collection in pararenal spaces, and ascites showed similar CT attenuation values (0-10 HU). The perinephric fluid was aspirated under ultrasound guidance. The aspirated fluid was white in color suggestive of chylous fluid. The analysis of aspirated fluid showed lymphocytes with elevated protein and triglycerides, features that were consistent with chylous fluid. No organism was isolated from the aspirated fluid. A diagnosis of bilateral renal lymphangiectasia and chronic parenchymal liver disease was made. The patient was managed conservatively.

Case 4

A 67-year-old male with a past medical history of myeloproliferative disease, liver parenchymal disease, and portal hypertension presented with persistent abdominal pain of one month's duration. A greyscale ultrasound scan of the abdomen showed features of right-sided hydronephrosis. The patient was further evaluated with a contrast-enhanced CT scan of the abdomen. The CT images showed a hypodense lesion measuring 5 x 3 cm in the right renal pelvis region. On post-contrast CT images, there was no demonstrable contrast enhancement of the lesion. The lesion was insinuating into the pelvicalyceal system, causing stretching of the calyceal system. There was no contrast opacification of the lesion seen even in the excretory phase. Few simple subcentimetric cysts were also seen in both kidneys. The imaging features were suggestive of right peripelvic renal lymphangiectasia. The patient was managed conservatively.

Case 5

**Figure 6:**
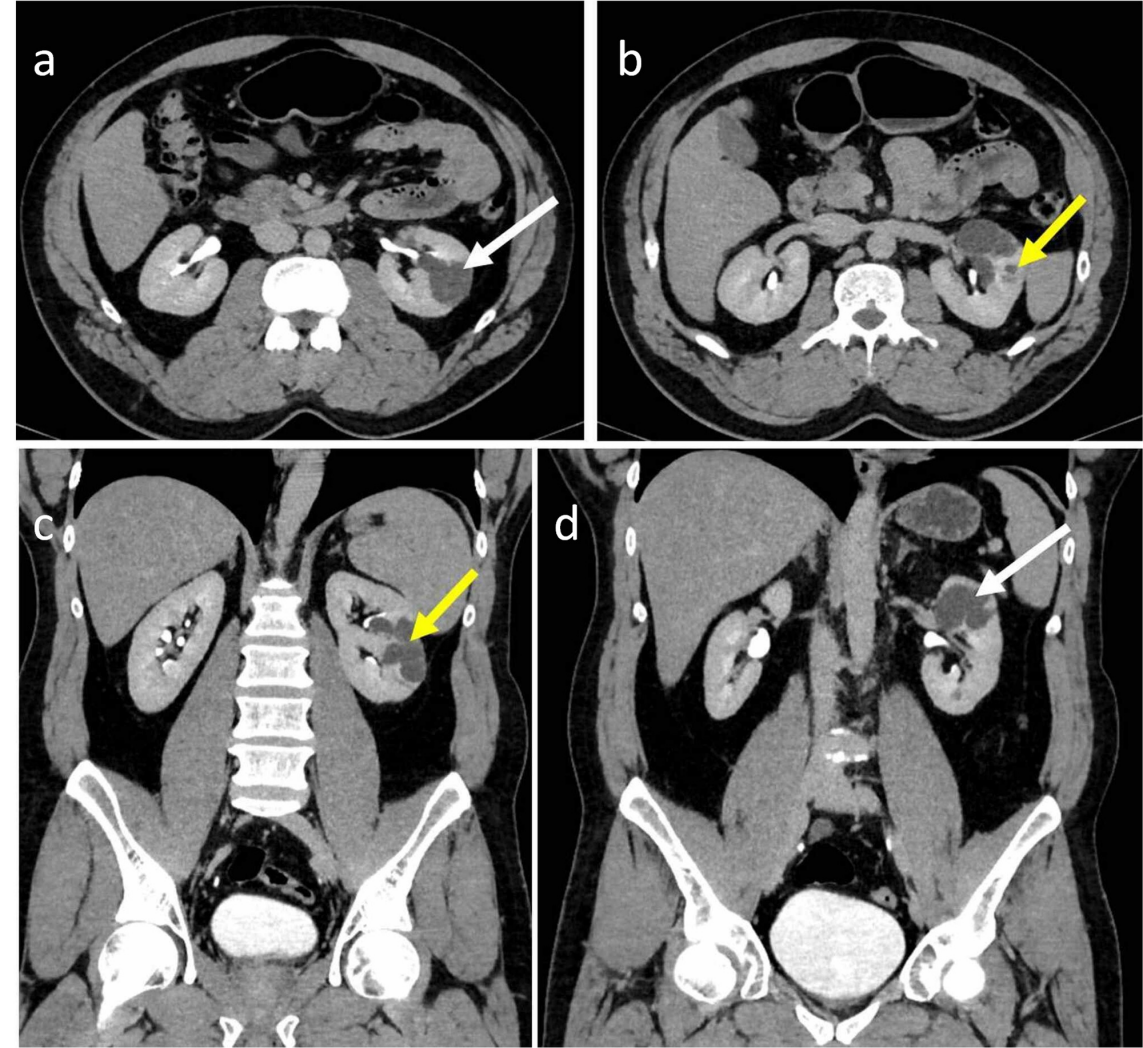
30-year-old male with left parapelvic and intrarenal lymphangiectasia Contrast-enhanced CT of the abdomen in the venous phase in axial (a, b) and coronal (c, d) planes demonstrates left parapelvic (white arrows) and intrarenal lymphangiectasia (yellow arrows) CT: computed tomography

A 30-year-old male presented with persistent left-sided abdominal pain for three months. The patient was evaluated with a contrast-enhanced CT scan of the abdomen. The CT images showed few hypodense lesions with fluid attenuation in the left renal parenchyma, and one of them was extending into the renal sinus (Figure 6). There was no significant enhancement in post-contrast immediate and delayed images. The rest of the visualized abdomen was normal. Because of the fluid attenuation of the cystic lesion with no post-contrast enhancement and since the lesions appeared to insinuate the pelvicalyceal system, a diagnosis of left parapelvic and intrarenal lymphangiectasia was made and the patient was managed conservatively.

In all these cases, patients were properly diagnosed on contrast-enhanced CT scans and other differential diagnoses were excluded. Prompt diagnosis of renal lymphangiectasia on CT images helped the referring physician in further management.

## Discussion

Pathophysiology and clinical features

One of the rare mimickers of cystic renal lesions is renal lymphangiectasia. Renal lymphatic malformation (RLM) is a benign entity and can be found in children and adults of both sexes and can be unilateral or bilateral. The incidence of RLM accounts for approximately 1% of all lymphangiomas [1]. The pathophysiology behind RLM is explained by the fact that the lymphatic drainage of the kidney, renal capsule, and the perinephric region intercommunicate through several large lymphatic trunks within the renal sinus. These lymphatics drain into the retroperitoneal nodes, namely paraaortic, paracaval, and inter-aortocaval lymph nodes. In renal lymphangiectasia, there is impairment in the drainage of larger renal sinus lymphatic trunks with resultant dilatation of peripelvic, and perinephric and intrarenal lymphatics [2]. Causative factors for RLM may encompass familial, developmental, and acquired spectrum [3].

Clinically, few patients are asymptomatic and the disease is diagnosed as an incidental finding; a few who are symptomatic have presented with abdominal pain, flank pain, abdominal mass, hematuria, proteinuria, and hypertension [4]. A few cases have also been reported with renal insufficiency and renal vein thrombosis [5].

Imaging appearances

The imaging appearances of RLM depend on the distribution and extent of lymphatics. The dilated lymphatics appear as cystic lesions in the perinephric, peripelvic, and intrarenal locations and produce typical imaging findings [6]. In some cases, ectatic lymph channels may also be distributed around the great vessels in the retroperitoneum [7]. Ascites is also noted in severe cases.

Ultrasound reveals anechoic cystic lesions in the renal sinus, known as peripelvic RLM, or in the perinephric regions, known as perinephric RLM, with or without septations [2,3]. Sometimes, cystic lesions are seen in the renal parenchyma, extending from there into the renal sinus. Another entity called intrarenal lymphangioma is a rare one and it can appear as a focal hyperechoic lesion in the renal cortex. In pediatric cases, it demonstrates enlarged kidneys with raised renal cortical echoes and loss of corticomedullary differentiation [8].

CT examination reveals cystic lesions showing fluid attenuation in the renal sinus (peripelvic or perinephric location) with or without septations. The CT attenuation value of an uncomplicated cyst measures between 0-10 HU. When there is a hemorrhage within the cyst, the CT attenuation value is increased and measures more than 60 HU. In contrast-enhanced CT, there is no opacification of cystic lesions on delayed scans in the excretory phase, which is an important feature that differentiates RLM from the dilated pelvicalyceal system [9]. In rare cases, RLM can also present as fluid collections in retroperitoneum due to dilated lymphatic channels. Unless complicated by hemorrhage, the fluid collections in all the spaces are clear on all imaging modalities [7].

On MRI scans, the cystic lesions appear hypointense on T1-weighted images and hyperintense on T2-weighted images. In the case of intrarenal lymphangiectasia, the kidney is enlarged in size with altered corticomedullary differentiation. In patients with impaired renal function, administration of iodinated contrast agents should be avoided. In those cases, MRI with MR excretory urography can be used as an alternative imaging technique for the evaluation of renal lymphangiectasia. T1-weighted MR excretory urography images acquired after giving intravenous gadolinium contrast agents can provide both anatomical and functional information [10]. MRI can detect thin septations within the cyst. In a few cases, retroperitoneal perivascular thin lymphatic channels may also be demonstrated.

Complications

The complications of RLM include renal vein thrombosis, renin-dependent arterial hypertension due to the compressive effect of the perirenal and peripelvic cysts, obstructive uropathy features due to compression of the collecting system by the larger cysts, intracystic hemorrhage, and superimposed infection [7,9].

Treatment and prognosis

Though the imaging features are typical, in a few cases, the confirmation is done by percutaneous aspiration of the cyst and by assessing the cytology of the aspirated fluid. The aspirated fluid of renal lymph differs from that of thoracic duct contents because renal lymphatic vessels are out of the pathway of lymphatic drainage of the mesentery. Thoracic duct contents are highly rich in fat and protein, whereas renal lymph contains mostly lymphocytes and small amounts of fat and protein [11]. Though there is a significant difference in the content of the renal and thoracic duct lymphatic fluid, any significant difference in the CT attenuation value is not yet reported in the literature.

RLM is a benign entity. Like other lymphatic lesions, lymphangiectasia can appear suddenly, grow rapidly, cease growth, or even regress spontaneously. Asymptomatic cases usually receive conservative management. But due to potential complications, especially renal failure and hypertension associated with RLM, periodic follow-up is necessary. Percutaneous aspiration of the collection is required in less severe symptomatic cases and also in cases presenting with pain on account of compression by the collection. However, the success rate is less in multiseptated larger lesions and leads to more recurrences. Laparoscopic ablation and nephrectomy are reserved for complicated cases and cases with multiple recurrences. However, nephrectomy is not advisable nowadays, as, in the case of asymmetrical bilateral involvement, the cysts in the contralateral kidney may increase in size [7,12].

A summary of the features of renal lymphangiectasia is provided in Table 1. 

**Table 1:** Summary of the features of renal lymphangiectasia RLM: renal lymphatic malformation

Features of renal lymphangiectasia	
Aetiology	Impairment in the drainage of larger renal sinus lymphatic trunks with resultant dilatation of intrarenal, peripelvic, and perinephric lymphatics
Incidence	RLM accounts for approximately 1% of all lymphangiomas
Gender ratio	No sex predilection
Age predilection	It can occur at any age
Risk factors	Any inflammation or obstruction causes blockage of lymphatic vessels. Some familial associations have also been described in the literature
Treatment	Asymptomatic cases receive conservative management. But due to potential complications, especially renal failure and hypertension associated with RLM, periodic follow-up is necessary. Percutaneous aspiration of the collection is required in less severe symptomatic cases and also in cases presenting with pain on account of compression by the collection. However, the success rate is less in multiseptated larger lesions and leads to more recurrences. Laparoscopic ablation and nephrectomy are reserved for complicated cases and cases with multiple recurrences. However, nephrectomy is not advisable nowadays, as, in the case of asymmetrical bilateral involvement, the cysts in the contralateral kidney may increase in size
Prognosis	It is a benign entity. Like other lymphatic lesions, lymphangiectasia can appear suddenly, grow rapidly, cease growth, or even regress spontaneously
Imaging findings	
Ultrasound	Anechoic cystic lesions in the renal sinus, or perinephric regions with or without septations. Intrarenal lymphangioma appears as a focal hyperechoic lesion in the kidney. Enlarged kidneys with raised renal cortical echoes and loss of corticomedullary differentiation
Computed tomography	Fluid attenuation in the renal sinus (peripelvic or perinephric location) with or without septations. In contrast-enhanced CT, there is no enhancement in early phases and there is no opacification of cystic lesions in the excretory phase
Magnetic resonance imaging	Cystic lesion appears hypointense on T1-weighted images and hyperintense on T2-weighted images. In contrast-enhanced T1-weighted images, there is no enhancement in the early phases. There is no opacification of cystic lesions in the post-contrast T1-weighted MR excretory urography images

Differential diagnosis

The differential diagnoses to be considered are polycystic kidney disease, hydronephrosis, multilocular cystic nephroma, urinoma, renal lymphoma, and nephroblastomatosis.

Polycystic kidney disease is a hereditary disorder characterized by multiple renal cysts and various systemic malformations. On ultrasound, the kidneys are massively enlarged with multiple well-defined cysts of varying sizes in the renal cortex replacing the normal renal parenchyma. On CT, multiple, well-defined, round, or oval-shaped cysts of varying sizes in the renal cortex with fluid attenuation are seen. There is no enhancement of the cysts on contrast-enhanced CT. In RLM, the renal cortex may show scalloping secondary to cysts in perinephric space. But in the case of polycystic kidney disease, the renal cortex shows multiple cysts with distortion of the renal parenchyma.

In hydronephrosis, there is dilatation of the collecting system, whereas RLM manifested as cystic lesions in the renal sinus often displace the otherwise normal collecting system. On contrast-enhanced CT, there is opacification of the collecting system on delayed scans, whereas, in RLM, there is no opacification of cyst on delayed scans in contrast-enhanced CT.

Multilocular cystic nephroma is a rare non-hereditary benign renal neoplasm arising from metanephric blastema. It is characterized by a focal multiloculated cystic mass of near-water HU ± herniating into the renal hilum. On ultrasound, it appears as a multicystic mass with no solid or nodular component. On CT, it appears as an encapsulated well-circumscribed mass with enhancing septa and no excretion of contrast agent into the cyst. At times, extension into the renal pelvis and ureter may also be seen. Even though imaging appearances are similar to RLM, they can be differentiated using contrast-enhanced ultrasound (CEUS). The enhancement pattern in the tissue between the cysts and loculi is the same as that of the normal renal parenchyma in lymphangioma due to the concept of septations that are normal renal parenchyma compressed between the focally enlarged lymph channels. However, in multilocular cystic nephroma, there is a malignant CEUS pattern rather than a normal parenchymal pattern [13].

Urinoma most commonly occurs due to blunt or penetrating renal trauma and also due to pelvi ureteric junction obstruction. Imaging on ultrasound and CT reveals a localized or diffuse cystic perirenal mass. However, urinary leakage is demonstrated on contrast-enhanced CT in the delayed phase, whereas in RLM, there is no evidence of contrast opacification of the cystic lesions in the excretory phase [6].

Renal lymphoma is characterized by multiple bilateral renal masses or perinephric involvement from retroperitoneal or renal spread. Ultrasound shows internal vascularity within the mass and showing soft-tissue attenuation with enhancement on contrast-enhanced CT. However, RLM is a cystic lesion (fluid attenuation) with no vascularity [14]. Associated features of retroperitoneal adenopathy, splenomegaly, or lymphadenopathy at other sites are also seen in the case of renal lymphoma.

Nephroblastomatosis is a rare pathologic process due to the presence of persistence embryogenic rests. On ultrasound, the major feature is enlarged diffusely hypoechoic kidneys. On CT, there are poorly enhancing soft tissue dense lesions intermingled with adjacent normally enhancing renal parenchyma. RLM shows fluid attenuation on imaging as opposed to soft tissue attenuation seen with nephroblastomatosis [14]. The salient features of differential diagnoses of RLM are presented in Table 2.

**Table 2:** Ultrasound and CT appearances of renal lymphangiectasia and its differentials CT: computed tomography: CEUS: contrast-enhanced ultrasound

	Ultrasound	CT
Renal lymphangiectasia	Anechoic cystic lesions in the renal sinus, or perinephric regions with or without septations. Intrarenal lymphangioma appears as a focal hyperechoic lesion in the kidney. Enlarged kidneys with raised renal cortical echoes and loss of corticomedullary differentiation	Cystic lesions showing fluid attenuation in the renal sinus or perinephric location with or without septations. In contrast-enhanced CT, there is no opacification of cystic lesions on delayed scans in the excretory phase. Presence of fluid collections in retroperitoneum due to dilated lymphatic channels
Polycystic kidney disease	Massively enlarged kidney with multiple well-defined cysts of varying sizes in the renal cortex replacing the normal renal parenchyma	Multiple, well-defined, round, or oval shape cysts of varying sizes in the renal cortex showing fluid attenuation unless it is not complicated. No enhancement of the cysts on contrast-enhanced CT
Hydronephrosis	Dilated collecting system due to obstructive causes	Opacification of collecting system on delayed scans. Able to identify the cause causing hydronephrosis; for example, calculus
Multilocular cystic nephroma	Focal multiloculated cystic mass with no solid or nodular component. On CEUS, the enhancement pattern in the tissue between the cysts and loculi exhibits a malignant CEUS pattern rather than a normal parenchymal pattern	Encapsulated well-circumscribed mass with enhancing septa and no excretion of contrast agent into the cyst. At times, extension into the renal pelvis and ureter may also be seen
Urinoma	Localized or diffuse cystic perirenal mass	Localized or diffuse cystic perirenal mass. Urinary leakage is demonstrated on contrast-enhanced CT in the delayed phase
Renal lymphoma	Multiple bilateral renal masses with internal vascularity are demonstrated on ultrasound	Multiple bilateral renal masses showing soft-tissue attenuation with enhancement on contrast-enhanced CT. Associated features of retroperitoneal adenopathy, splenomegaly, or lymphadenopathy at other sites are also seen in the case of renal lymphoma
Nephroblastomatosis	Enlarged diffusely hypoechoic kidneys	Poorly enhancing soft tissue density lesions intermingled with adjacent normally enhancing renal parenchyma

## Conclusions

On imaging, RLM is characterized by cystic lesions in the renal sinus or perinephric location with no features of contrast opacification of the cystic lesions on early as well as delayed scans in contrast-enhanced CT. The radiologist should be familiar with the findings and different forms of imaging appearances and must be able to help the referring clinician with the correct information required to decide the appropriate treatment for each individual and thereby avoid unnecessary invasive procedures for a benign cause.
